# Research Progress in the Degradation of Chemical Warfare Agent Simulants Using Metal–Organic Frameworks

**DOI:** 10.3390/nano14131108

**Published:** 2024-06-28

**Authors:** Taotao Huang, Qian Chen, Hui Jiang, Kui Zhang

**Affiliations:** 1 State Key Laboratory of NBC Protection for Civilian, Beijing 102205, China; 2 School of Chemistry and Chemical Engineering, Anhui University of Technology, Ma’anshan 243032, China; 18356134730@163.com (T.H.); cqde2007@ahut.edu.cn (Q.C.)

**Keywords:** metal–organic framework, chemical nerve agent, simulant, degradation

## Abstract

Chemical warfare agents primarily comprise organophosphorus nerve agents, saliva alkaloids, cyanides, and mustard gas. Exposure to these agents can result in severe respiratory effects, including spasms, edema, and increased secretions leading to breathing difficulties and suffocation. Protecting public safety and national security from such threats has become an urgent priority. Porous metal–organic framework (MOF) materials have emerged as promising candidates for the degradation of chemical warfare agents due to their large surface area, tunable pore size distribution, and excellent catalytic performance. Furthermore, combining MOFs with polymers can enhance their elasticity and processability and improve their degradation performance. In this review, we summarize the literature of the past five years on MOF-based composite materials and their effectiveness in degrading chemical warfare agents. Moreover, we discuss key factors influencing their degradation efficiency, such as MOF structure, pore size, and functionalization strategies. Furthermore, we highlight recent developments in the design of MOF–polymer composites, which offer enhanced degradation performance and stability for practical applications in CWA degradation. These composite materials exhibit good performance in degrading chemical warfare agents, playing a crucial role in protecting public safety and maintaining national security. We can expect to see more breakthroughs in the application of metal–organic framework porous materials for degrading chemical warfare agents. It is hoped that these innovative materials will play a positive role in achieving social stability and security.

## 1. Introduction

**Figure 1:**
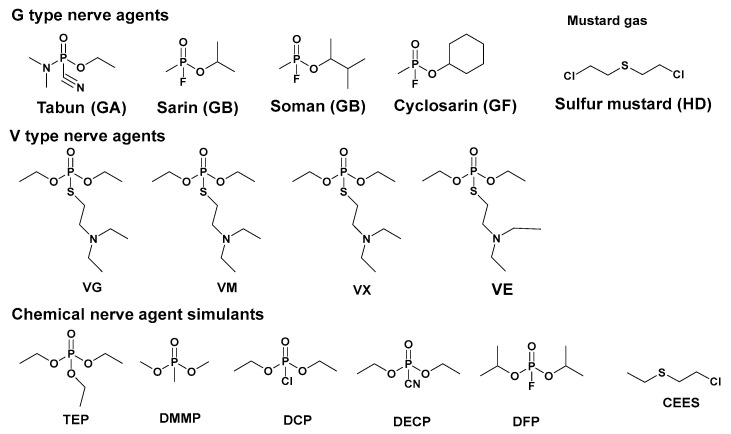
Chemical structures of CWAs and their simulants.

Chemical warfare agents represent a formidable threat due to their highly toxic nature, capable of causing severe disabling or lethal effects on the human body [1,2,3]. These agents encompass a diverse array of substances, including nerve agents, corrosive agents, and asphyxiating agents. In recent years, nerve agents have been used in various conflicts, such as the Iraq War in 1988 [4], the Syrian War in 2013 [5], and the Tokyo subway incident in 1995 [6]. Notable incidents include the assassination attempt using the VX nerve agent in Kuala Lumpur in 2017 and the Novichok nerve agent attack in the United Kingdom in 2018 [7]. Chemical warfare agents consist of various types of substances, including irritants, nerve agents, hepatotoxic substances, and suffocating substances, capable of causing burns and ulcers on the skin and mucous tissues. The development of organophosphorus nerve agents was initially serendipitously discovered by German chemist Gerhard Schrader during his investigation into novel insecticides. Organophosphorus nerve agents are primarily classified into two categories known as G and V agents. Tabun (GA) is one of the earliest nerve agents developed and was first synthesized in Germany during World War II. It is an oily liquid with a slightly fruity odor. Tabun can be absorbed through inhalation, ingestion, or skin contact, making it extremely dangerous. Sarin (GB), another well-known G-type agent gained international recognition after being used in several high-profile incidents. This colorless and odorless liquid has been associated with acts of terrorism due to its ability to cause rapid paralysis of the respiratory system when released as a gas. Soman (GD) has a moderate vaporization rate, making it a medium vaporization toxin that is easy to disperse [8]. Organophosphorus nerve agents disrupt regular neurotransmitter transmission, resulting in symptoms such as shock, seizures, paralysis, coma, and, in severe cases, death [9,10,11,12]. Moreover, these agents pose significant risks to the respiratory system and can cause irreversible damage to the nervous system, highlighting the importance of detection and degradation technologies. The main types of organophosphorus nerve agents and simulants are listed in Figure 1. Gaseous forms of nerve agents exist at room temperature under atmospheric pressure, making them easily dispersible and posing a significant threat to humans. Liquid forms include sarin and cyanides, which are typically used in liquid form due to their ability to contaminate environments over large areas, posing a serious hazard to both humans and ecosystems alike. Mustard gas is released in foam form and enter the body through the eyes, skin, respiratory tract or gastrointestinal tract, causing immense damage such as blistering of the skin, visual abnormalities or blindness, respiratory failure, and even death [13,14,15].

Various catalysts have been developed for degrading chemical warfare agents, including metal oxides, metal-organic frameworks (MOFs) [16,17,18,19,20], polyoxometalates (POMs) [21,22,23], and porous organic polymers [24,25,26,27]. These catalysts exhibit a promising performance in the degradation of nerve and blowing agents. Despite extensive research aimed at enhancing the performance of catalysts in degrading chemical warfare agents, several challenges persist. For instance, some catalysts have limited degradation capabilities, operate under harsh catalytic conditions, and are difficult to recover. The development of efficient degradation catalysts aims to address these issues by providing protective digestion for a wide range of chemical warfare agents, reducing reliance on external auxiliary conditions, and enabling catalyst reusability.

In recent years, MOFs or porous coordination polymers (PCPs) composed of metal ions have emerged as a novel class of porous materials [28,29,30,31]. MOFs are crystalline coordination polymers characterized by their open scaffolds, which possess inherent voids for potential applications [32,33,34]. These structures consist of metal ion/cluster-based nodes, commonly referred to as secondary building units (SBUs), which are interconnected through coordination bonds with organic linkers primarily comprising carboxylic acids. Due to the inherent variability of metals and linkers, MOFs exhibit diverse structures and properties, characterized by exceptional porosity and surface areas typically exceeding 1000 mg. These remarkable attributes render MOFs highly promising candidates for applications in gas storage and separation, catalysis, drug delivery, sensing removal of harmful compounds, light harvesting, and photocatalysis [35,36,37,38,39]. Porous MOFs are intricate three-dimensional crystalline structures formed through the coordination of metal ions and ligands, exhibiting complex architectures, adjustable pore sizes, and substantial specific surface areas. The designability and controllability of MOFs pore surfaces render them highly promising for diverse applications encompassing substance separation adsorption, and catalysis [40,41,42].

Consequently, MOFs have garnered significant attention from researchers worldwide. Extensive investigations have been conducted on nano-doping techniques applied to both MOFs and polymers. In most cases, polymer chains are typically entangled randomly, leading to properties that are predominantly influenced by the average behavior of these disordered multichains. For polymer encapsulation systems, the adjustability and tunability of MOF nanochannels play a crucial role in achieving precise control over various aspects, such as the polymer chain number, polymer environment, and chain orientation. The controlled integration of polymers and MOFs can lead to the development of advanced functional composites that exhibit properties surpassing those of their components. To emphasize the significance of this field, we analyzed an increasing number of research papers published between 2019 and 2023, focusing on MOFs and polymer–MOF hybridization. This review paper provides an overview of recent research advancements in MOF/polymer functional materials for degradation and protection against chemical warfare agents.

## 2. Degradation Mechanism of Chemical Warfare Agents

**Figure 2:**
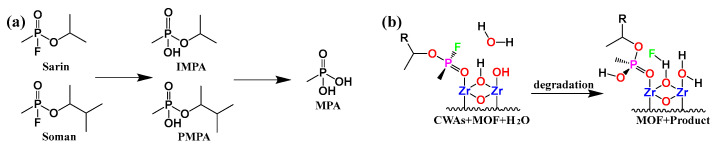
(**a**) The degradation of GB and GD. (**b**) The degradation between MOFs and CWAs.

Nerve agents are primarily categorized into G and V agents, with hydrolysis being the primary method of detoxification [43,44,45]. The detoxification process of Class G agents primarily involves nucleophilic substitution. For instance, both Soman (GD) and Sarin (GB) undergo hydrolysis via the nucleophilic substitution mechanism. GB hydrolyzes to yield hydrofluoric acid and non-toxic isopropyl methyl phosphonic acid (IMPA), while GD hydrolyzes to produce hydrofluoric acid and pinacolyl methylphosphonic acid (PMPA). PMPA can further react with water to form methylphosphonic acid (MPA). The hydrolysis of certain organophosphorus nerve agents generates different intermediates depending on the pH conditions, as depicted in Figure 2a. Regardless of the pH conditions, the final products obtained from hydrolysis are consistently phosphoric acid. The hydrolysis of P-F by Sarin and Soman is a critical process in understanding the degradation of nerve agents with MOFs. For example, the mechanism of nerve agent hydrolysis is illustrated in Figure 2b. Zr-MOFs have strong Lewis acids that are capable of reacting with nerve agents in the presence of a protic solvent [46]. 

**Figure 3:**
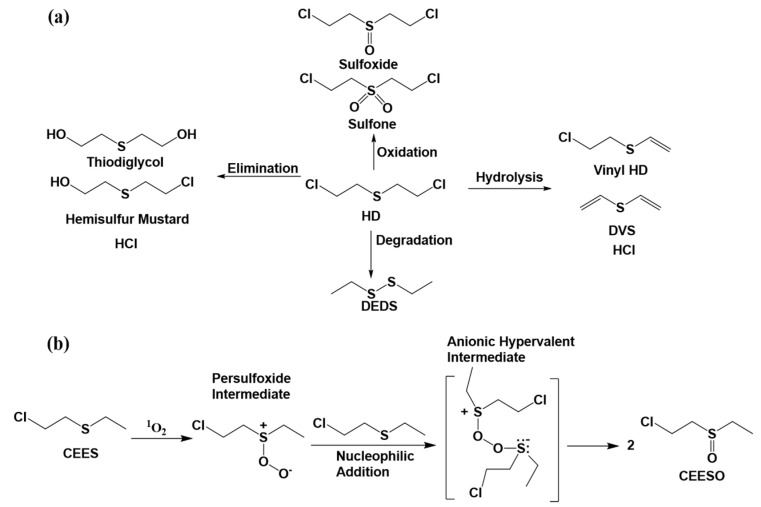
(**a**) The possible degradation process of mustard gas (HD). (**b**) The oxidation between MOFs and CEES.

The detoxification mechanisms for mustard gas (HD) involve four degradation pathways. HD can undergo single-molecule nucleophilic substitution reactions, resulting in the loss of hydrogen chloride and the formation of non-toxic products such as vinyl HD (VHD) and diethylene vinyl HD (DVHD or DVS). This pathway effectively converts harmful hydrogen chloride into non-toxic compounds, with both VHD and DVHD exhibiting lower toxicity levels, as illustrated in Figure 3a, showing the possible degradation process of mustard gas (HD). The recombination pathway involves the breaking of S–C bonds to form ethyl sulfide radicals, which then undergo radical reactions to recombine into diethyl disulfides. The third pathway is hydrolysis, where sulfonium intermediates undergo hydrolysis to yield half-sulfur mustard and thiodiglycol, the latter of which finds application as an anticancer drug. However, the strong hydrophobic nature of HD limits its dissolution rate in water. It is crucial to carefully select mild oxidants for this reaction, as excessive oxidation may generate more toxic substances. Under irradiation, the singlet oxygen was produced by MOFs materials as shown in Figure 3b. The reaction mechanism was illustrated for photooxidation of CEEES using MOFs materials.

In summary, understanding the mechanisms underlying the detoxification of HD holds significant implications for improving environmental pollution control efficiency, safeguarding human health, and advancing the development of protective equipment. Therefore, careful consideration of appropriate conditions during practical applications is imperative to ensure safety and efficacy.

## 3. MOFs Degradation of Chemical Warfare Agents

MOFs represent a class of porous crystalline materials comprising metal ion units or clusters linked by robust coordination bonds with organic ligands. Their remarkable features, including high surface area, permanent porosity, and diverse topological properties, render MOFs highly attractive for a wide range of applications, including sensing, drug delivery, and catalysis. Functional sites within MOFs can be introduced through pre- or post-functionalization using organic ligands, inorganic metals, and various functional moieties. Furthermore, enzymes, metal nanoparticles (NPs), and organometallic compounds can be employed to further enhance the functionalization of MOFs. As previously discussed, the versatility of MOFs, enabled by functionalization on ideal surfaces and within porous environments, positions them as promising candidates for research on the degradation of chemical warfare agents.

Singlet oxygen is the singlet excited state of oxygen, which has significantly different chemical reactivities compared to the kinetically inert triplet ground state of oxygen [47]. The first MOF selected by Farha et al. for the generation of singlet oxygen was PPCN-222. Its organic linkers possess porphyrin photosensitivity, and the three-dimensional structure of PPCN-222 increases the distance between the linkers, enabling the separation and heterogenization of porphyrin molecules [48]. Therefore, the problem of reduced singlet oxygen production due to aggregation and degradation of free porphyrin molecules in the solution is eliminated. 

Nevertheless, the MOFs are impeded by their crystalline powder form, posing considerable obstacles during the processing phase. This limitation has sparked an intensified research effort toward composite materials. To overcome this challenge, the primary avenue has been explored by embedding MOFs within organic polymers. MOFs have high stability due to the strong Lewis acid metal nodes with metal ions as the center. MOFs are typically synthesized using hydrothermal or solvothermal methods, where crystals gradually form from a hot solution. Throughout this process, organic ligands retain their original functionality, ensuring it is preserved within the framework. At present, MOF materials are widely used for practical applications in protective equipment. 

### 3.1. Chemical Nerve Agent Simulants with MOFs

Building upon this foundation, subsequent investigations have expanded the repertoire of MOFs to encompass promising capabilities in combating CWA threats. Further exploration has revealed that Cu-MOF (NENU-11) exhibits analogous activity to hydrolyzing nerve agent analogs, suggesting its potential as a formidable candidate in CWA defense strategies [49]. Additionally, derivatives of MIL-101 modified with dialkylaminopyridines (DAAPs) have emerged as contenders with reported hydrolytic activity toward nerve agent simulants [50]. Hydrolysis of paraoxon has been achieved by DAAP-MOF in a water/acetonitrile mixture (100% conversion after 24 h at pH 10). Compared to the parent MIL-101 or DAAP materials, MIL-101 MOF modified with DAAP ligands can improve the catalytic activity, which is of great significance for environmental detoxification and defense applications. However, despite their inherent potential, challenges such as poor air/water stability and lower catalytic activity impede their efficacy in serving as efficient catalysts for decomposing nerve agents and their simulants. The field of MOF synthesis has embarked on the exploration of novel structural types, embraced diverse strategies for modifying the structure and properties of MOFs, and delved into a multitude of functions and applications. The catalytic activity of MOF can be manifested as either link- or cluster-mediated catalytic activity.

**Figure 4:**
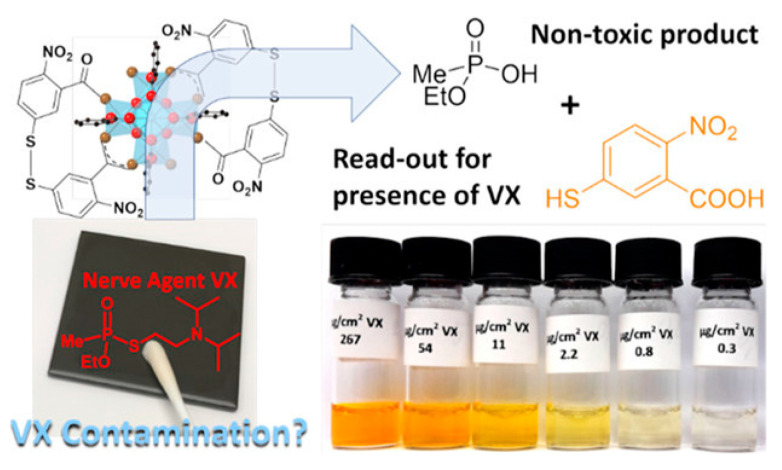
The structure of NU-1000 and the colorimetric detection of VX [51].

In 2019, Martin et al. designed the NU-1000 material to detect and degrade the nerve agent VX. NU-1000 can hydrolyze VX into non-toxic phosphoric acid and mercaptan 2- (diisopropyl amino) ethyl mercaptan, as shown in Figure 4. In this study, four coordination sites exist for each Zr element of MOFs, which can be functionalized with two carboxylic acids in 5,5′-dithiobis (2-nitrobenzoic acid) (DTNB) [51]. This dimeric compound reacts efficiently with the sulfhydryl group via S–S bond exchange and releases the chromophore RS-H. Therefore, (DTNB)@NU-1000 can degrade VX through noticeable color changes, proving that the chemical warfare agent is effectively degraded, and the degradation time is 20 min.

**Figure 5:**
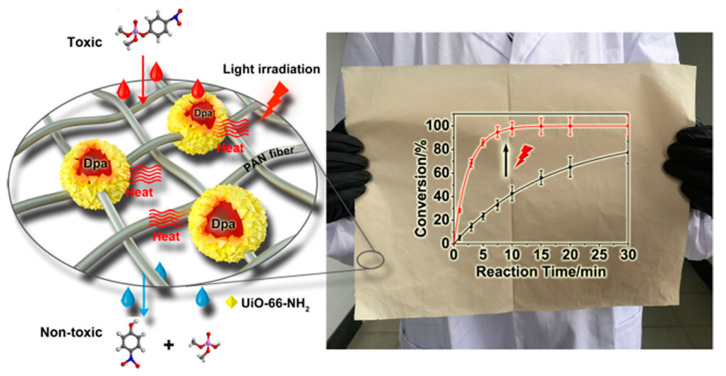
Schematic illustration of the process of dopamine modification of UiO-66-NH_2_ and degradation of dimethyl 4-nitrophenyl phosphate (DMNP) [52].

In 2019, Li et al. used dopamine–melanin (Dpa) nanoparticles as the inspired biological template to prepare the Zr-organic framework UiO-66-NH_2_, as shown in Figure 5. The designed Dpa@UiO-66-NH_2_ can increase the turnover frequency (TOF) of the nerve agent analog DMNP by 2.9 and 1.7 times, respectively, under a near-infrared laser and simulated sunlight irradiation conditions [52]. Dpa@UiO-66-NH_2_ was further modified into polymer fibers by electrospinning, and the degradation rate exceeded that of pure MOF powder for the first time, with a half-life of 1.8 min. Dpa@UiO-66-NH_2_ fabrics can be prepared on a large scale and have the potential to be the next generation of gas mask materials as stand-alone gas filters. This study addressed the persistent challenge of diminished catalytic efficacy in MOF textiles, exhibiting superior detoxification capabilities compared to pure MOF powders. The innovative approach of photothermally enhanced catalytic detoxification holds potential for transferability to other catalytic detoxification systems, which offers promise for CWA purification technologies and next-generation gas masks.

**Figure 6:**
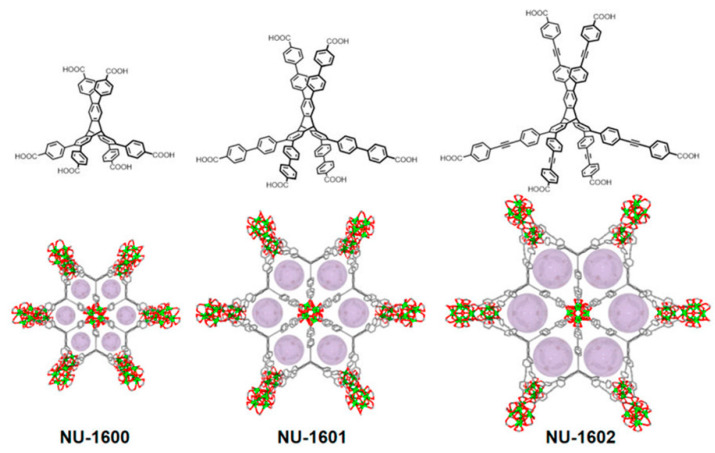
Schematic diagram of NU-1600, NU-1601, and NU-1602. Atom color scheme: Carbon (C) is depicted in gray, Zirconium (Zr) in bright green, and Oxygen (O) in red. Hydrogen atoms are omitted for clarity [53].

In 2019, Chen et al. designed and synthesized a series of zirconium MOFs with defects, namely, the NU-160X series (X = 0, 1, 2) with microporous structures, as shown in Figure 6. The BET was 2085 m^2^/g, and the adsorption isotherm by N_2_ at 77 K showed that the pore capacity was 0.82 cm^3^/g [53]. Zr-MOF showed efficient elimination activity against nerve agents and their simulators in the presence of liquid and solid bases. This study provides a reference for the rational design and synthesis of multifunctional high-connectivity materials.

**Figure 7:**
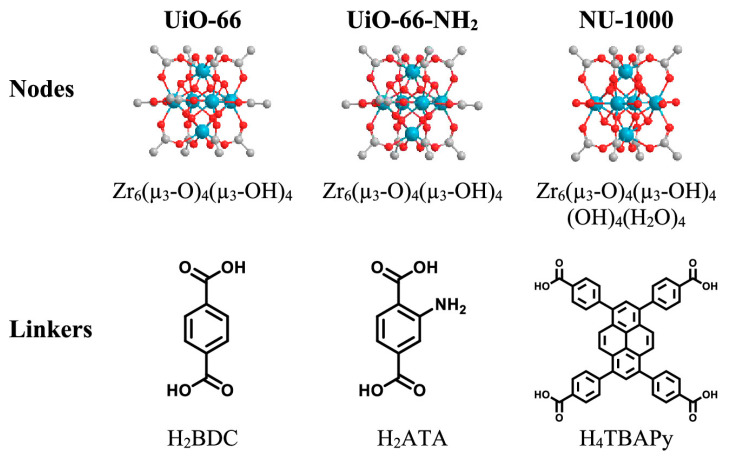
Detailed structures of the nodes and linkers of Zr-MOFs. Atom color scheme: Zirconium (Zr) is depicted in blue, Oxygen (O) in red, and Carbon (C) in gray. Hydrogen atoms are omitted for clarity [55].

In 2019, Kie et al. developed a simple and scalable synthesis method for Zr(IV)MOF(UiO-66) to improve its catalytic performance by endorsing the catalyst with abundant active sites [54]. At the same time, 4-ethylmorphazine (4-EM) and polyethylenimine (PEI) were used to degrade nerve agent simulants. Three synthesized UiO-66 series catalysts were characterized and applied to nerve agent simulators (methylphosphate, MPO) to test the hydrolysis reaction rate. Additionally, a reaction mechanism for nucleophilic offensive degradation based on hydroxyl functional groups was proposed. In 2019, Wang et al. found that the hydrolytic activity of UiO-66 on CWAs increased significantly with an increase in water content [55]. Specifically, the initial hydrolysis rate of UiO-66 for methyl paraoxon increased from 6 μmol at a 0 wt% water load to 140 μmol at a 400 wt% water load. They designed a method for solid-state degradation analysis and quantitative elimination for the first time, as shown in Figure 7.

In 2019, Jared et al. successfully synthesized amino-modified Zr_6_ single-component heterogeneous catalysts using the solvent-assisted linker introduction method (SALI) and applied them to the hydrolysis reaction of organic phosphorus compounds [56]. Under pure water conditions, this material can efficiently degrade DMNP and nerve agent VX. Modulators play a crucial role in regulating crystal orientation and particle size while affecting the quantity of the connecting ligands or missing nodes during the MOF synthesis process. Employing the SALI technique for fabricating amino-modified Zr-MOFs effectively degrades organic phosphorus compounds under no-buffer solution conditions.

**Figure 8:**
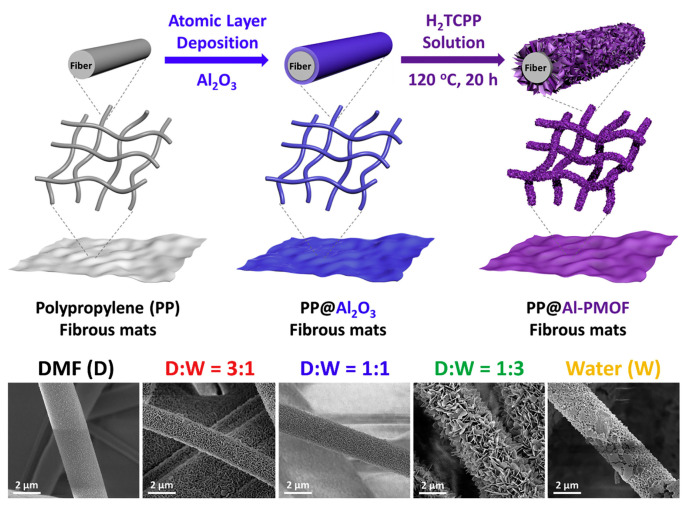
The synthesis of Al-PMOF/fiber textiles [57].

In 2020, Lee et al. reported the exceptional photocatalytic activity of metal-rich Al (OH)O_4_ clusters composed of Al-PMOFs (Al-porphyrin-based MOFs) toward the toxic sulfur mustard mimetic 2-chloroethyl ethyl sulfide (CEES) under visible light irradiation for the first time [57]. The Al-PMOF photocatalyst exhibited promising potential for generating reactive singlet oxygen under LED irradiation, leading to rapid and selective oxidation to harmless CEESO. Furthermore, this paper demonstrated the successful immobilization of Al-PMOF films on polymer nonwoven fabrics. Additionally, a well-controlled conversion of an Al_2_O_3_ solid film using dimethylformamide/water cosolvent effectively immobilized Al-PMOF within the polymer, as illustrated in Figure 8. The surface-incorporated Al-PMOF film exhibited an exceptionally rapid CEES detoxification turnover frequency on a per unit mass of MOF basis, surpassing the highest-efficiency MOF powder reported to date by more than 10 times and outperforming the MOF film with the best-reported degradation efficiency by 2 times.

Although zirconium–organic frameworks (Zr-MOFs) are already one of the fastest catalysts for nerve agent hydrolysis, the practical application of MOF/fiber composites still needs to be improved due to a lack of reliable methods to integrate them into fiber supports. In 2020, Li et al. designed a dopamine-modified MOF material that uses dopamine (PDA) as a mediator for the preparation of Zr-MOF(UiO-66-NH_2_) nanofibers to achieve photothermal catalytic degradation of CWA simulators. UiO-66-NH_2_ nanocrystals with a high load and good adhesion can be prepared on polyamide nanofibers pre-coated with a PDA layer. When exposed to simulated sunlight, the half-life of PA-6@PDA@UiO-66-NH_2_ nanofibers for the hydrolysis of DMNP, a nerve agent simulator, was only 0.5 min [58]. Compared to the reported UiO-66-NH_2_ powder and UiO-66-NH_2_/fiber composites, the degradation efficiency was significantly improved.

**Figure 9:**
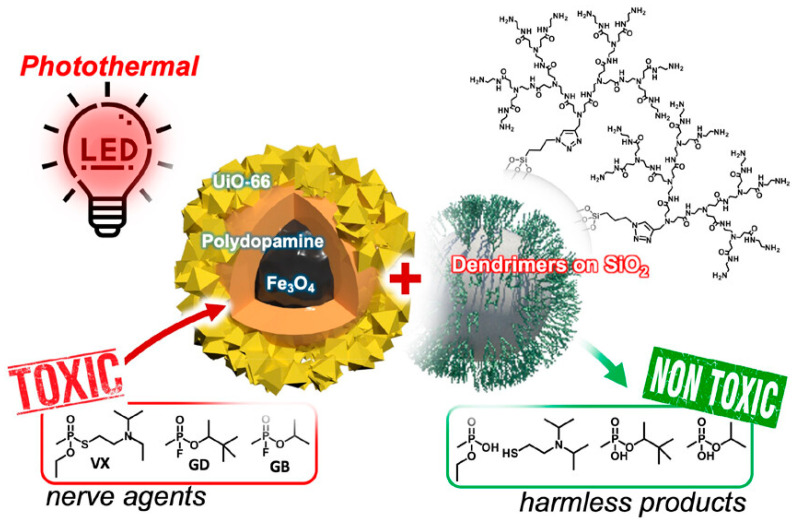
Illustration of the degradation of nerve agents using Fe_3_O_4_@PD@UiO-66 [59].

Generally, catalysts based on MOFs typically require alkaline reaction media to achieve rapid hydrolysis. N-ethylmorpholine (NEM) is commonly used to restore the activity of metal reaction centers in MOFs. However, these alkaline conditions can significantly decrease the stability of MOFs, leading to structural collapse and difficulties in maintaining catalytic activity and reusability. In 2023, Lee et al. developed a novel photo-thermal active catalytic composite material, Fe_3_O_4_@PD@UiO-66 for decomposing chemical warfare agents [59]. By introducing iron oxide nanoparticles, rapid recovery can be achieved after the reaction. As shown in Figure 9, the polydimethylsiloxane (PD) layer converts absorbed light energy into heat energy and utilizes branched polymer-functionalized silica particles (NH_2_-DS) as a heterogeneous catalyst regenerator instead of N-ethylmorpholine, enabling the fast degradation of sarin (GB), soman (GD), and nerve agent VX with half-lives of 4.2, 8.7, and 14.0 min, respectively.

### 3.2. MOF Degradation of Mustard Gas Simulants

**Figure 10:**
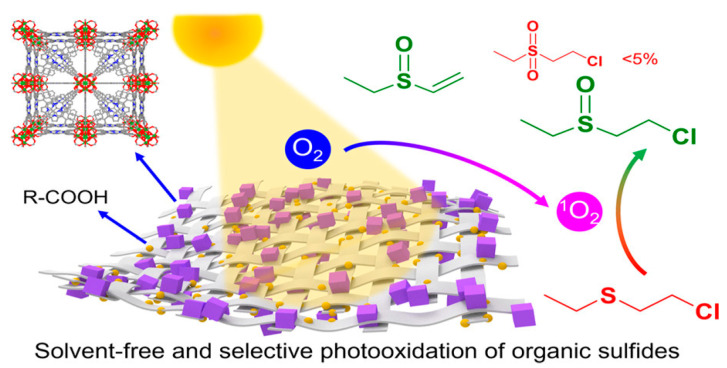
Illustration of MOF fibers’ decomposition of CEES [68].

Dichlorodiethyl sulfide has high hydrophobicity, making it very slow to detoxify by dechlorination under natural conditions. Dichlorodiethyl sulfide can undergo oxidation to sulfoxide, which significantly reduces toxicity. However, the complete oxidation of dichlorodiethyl sulfide can yield a highly toxic product called sulfone, which is considerably more hazardous than sulfoxide due to its increased reactivity and potential to cause severe health issues. Therefore, meticulous control over the oxidation process is crucial to ensure partial oxidation to prevent the production of the highly toxic sulfone compound [60,61,62]. CEES reacts with singlet oxygen to form a persulfoxide molecule that is longer-lived than singlet oxygen [63,64,65]. For example, under purple irradiation, NU-100 containing pyridyl organic junctions can produce singlet oxygen. MOFs and their derivatives have demonstrated efficient selective oxidation of toxic organic sulfides, such as CEES and or mustard gas, under visible light or ultraviolet radiation [16,66,67]. However, the requirement for specific solvents like methanol to achieve satisfactory selectivity limits the practical application of MOFs in protective equipment. In 2021, Liu et al. reported the development of a series of MOF/textile composite materials containing multiple hydrogen bond donors that enable solvent-free selective oxidation of organic sulfides, as depicted in Figure 10. Remarkably, with a catalyst loading of only 3 mol%, complete decomposition of all CEES was achieved within 15 min, with a reaction half-life of 4.4 min, along with excellent stability and reusability. In contrast, the original MOF-525 powder converted merely 30% of the CEES after 15 min under identical conditions [68].

In 2020, Bandosz et al. prepared UiO-66 with nanographene or oxidized graphitic nanospheres, which was applied to the degradation of CEES from a vapor phase [69]. The MOFs were tested using BET with a maximum weight of 632 mg/g, higher absorption, and catalytic degradation than UiO-66. UiO-66 composite materials were employed in conjunction with either graphene or nitrogen-doped carbon–graphene nanospheres as composites for the degradation of CEES in the gas phase. These composites exhibited a remarkable enhancement in both degradation and adsorption properties. The results revealed that the catalytic activity was attributed to the interfacial defects between the modifier and the MOF, which served as active sites for the reaction. Furthermore, alterations in surface morphology and void size of the composite influenced both the reaction site activity and the MOF dispersion. The incorporation of graphite nanoparticles or oxidized graphitic carbon nitride nanospheres (~10 wt%) into UiO-66 composites led to a significant enhancement in the detoxification efficiency of CEES vapors by these materials, both in terms of adsorption capacity and surface reactivity, when compared to pristine UiO-66. The activity of this modifier can be attributed to the novel interface between MOF units/defects, which generates active sites through the reaction between zirconium clusters and surface groups of the modifier that act as MOF linkers. Furthermore, alterations in morphology and porosity were also observed alongside the creation of new catalytic centers. These findings suggest that the mechanism underlying CEES gas–solid interactions for detoxification differs from that in CEES liquid/dissolved liquid–solid interactions. In the gas phase, the cleavage, dehydrohalogenation, and oxidation pathways predominate over hydrolysis. Therefore, the enhanced performance of nanocomposites can be attributed to the incorporation of defect sites and the effective dispersion of active sites.

MOFs exhibit exceptional efficacy in the adsorption and detoxification of carbohydrates, owing to their remarkable porosity and adjustable reactivity. MOFs can be employed as tailored adsorbents with high affinity toward these compounds, enabling rapid degradation of chemical warfare agents (CWAs). In 2020, Farha et al. conducted a comprehensive investigation targeting GBs and CEES, encompassing NU-1000, dehydrated NU-1000, benzoic acid-covered NU-1000, NU-901, MOF-808, NU-1008, MOF-525, NU-1200, UiO-66, and defective UiO-66 [70]. Through subtle variations in MOF design, they systematically investigated the impact of surface area/pore volume, ligand selection, and pore functionalization on the capture and catalytic degradation performance of CWAs. They employed differences in surface area/pore volume, SBU connectivity, pore functionalization, and open metal sites to probe the adsorption behavior toward sarin gas and 2-chloroethyl ethyl sulfide as a mustard gas mimetic. For instance, NU-1000 exhibited exceptional adsorption capacity and reactivity, rendering it an excellent candidate for adsorption filtration materials. Conversely, UiO-66 and MOF-808 demonstrated remarkable nerve agent degradation capabilities. The increased surface area, pore volume, and enhanced hydrogen bonding interactions contribute to the elevated uptake of both GB and CEES. UiO-66, defective UiO-66, and MOF-808 exhibited the highest reactivity toward GB due to a higher density of active sites per unit volume. These findings provide valuable structure–property relationship data for designing novel degradable MOF materials.

In 2020, Liu et al. successfully synthesized two novel polyoxovanadate-based MOFs, namely V-Co-MOF and V-Ni-MOF, under mild hydrothermal conditions [71]. This study investigated the catalytic activity of these compounds toward the oxidation of CEES, an analog of mustard gas. The experimental results demonstrated the ability of V-Co-MOF and V-Ni-MOF catalysts to facilitate the oxidation of CEES, yielding 2-chloroethyl ethyl sulfoxide (CEESO) as the sole non-toxic product. Notably, V-Co-MOF exhibited superior catalytic activity, achieving complete conversion of CEES within 10 min, whereas V-Ni-MOF only converted 47.5% of CEES under identical conditions. Investigation into the catalytic reaction mechanism revealed that the exceptional performance of V-Co-MOF can be attributed to the synergistic interactions between two active sites: the oxidant H_2_O_2_ interacts with the V site, resulting in the formation of a highly toxic product. The V site facilitates the generation of vanadium peroxide with high oxidation activity. The S atom coordinates with the tetra-coordinated Co(II) center to form 2-chloroethyl ethanesulfonic acid cation, which enhances the ease of CEES oxidation to CEESO according to the vanadium peroxide oxidation mechanism. This coordination also reduces the molecular size distance between CEES and the obtained vanadium peroxide, significantly improving the rate of catalytic reaction. This study presented the pioneering utilization of a dual active site polymetallic oxonate-based MOF catalyst for effectively facilitating the oxidative detoxification of CEES, thereby offering valuable insights into the rational design of highly efficient MOF-based catalysts for enhanced CEES purification.

Zhao et al. prepared zirconium-based MOF materials through solvothermal assembly [72]. By employing a solvent–thermal assembly technique utilizing triphenylamine tricarboxylic acid ligands and Zr(IV) salts, they successfully synthesized a zirconium-based MOF. Structural simulations revealed that this framework exhibits a two-dimensional layered structure, with its inorganic building units comprising relatively uncommon 12-nuclear Zr_12_ clusters. The inherent photoresponsive properties of the photochromic triphenylamine moiety, combined with its exceptional stability, make the synthesized MOF a highly efficient heterogeneous photocatalyst for sulfide oxidation, which is a crucial reaction in both the environmental and pharmaceutical sectors. The photocatalytic activity of the synthesized MOF was initially investigated under various conditions using thioanisole as a representative sulfide substrate. Upon exposure to blue light, the MOF exhibited remarkable efficiency and selectivity for the aerobic oxidation of thioanisole in methanol for 10 h, utilizing molecular oxygen from ambient air as the oxidant. Although the system exhibited commendable photocatalytic performance toward sulfide substrates, various thioanisole derivatives, and even a sulfur-containing nerve agent mimic (2-chloroethyl ethyl sulfide), its catalytic duration is prolonged and necessitates an oxygen-rich environment for effective implementation, thereby limiting its potential for further application in photocatalytic degradation.

In 2021, Li et al. reported a series of MOFs MTCPP-La based on metal tetrakis(4-carboxyphenyl)porphyrin and La(III), which was employed as a heterogeneous photocatalyst for the selective oxidation of CEES [73]. Under the irradiation of blue light-emitting diodes (LEDs), M-TCPP-La generates superoxide ions and singlet oxygen, thereby participating in the selective oxidation of CEES to form CEESO. The metal–organic backbones (M-TCPP-La) were synthesized through solvothermal conditions by assembling La(III) ions, transition metal ions (Fe^2+^, Mn^2+^, Ni^2+^, Co^2+^, and Zn^2+^), and TCPP ligands. These 3D porous structures with 1D channels and La-carboxylic acid rod SBUs exhibited remarkable thermal and chemical stability, along with excellent photocatalytic activity in the selective oxidation of CEES for CEESO preparation. Among them, the Fe-TCPP-La photocatalyst exhibited the shortest half-life of 2.5 min for CEES degradation. Mechanistic studies revealed that both O_2_^•−^ and ^1^O_2_ were generated and actively involved in the oxidation of M-TCPP-La by CEES. The present study demonstrated, for the first time, the utilization of superoxide ions for the degradation of mustard mimics, thereby introducing a novel oxidant for rapid and efficient detoxification. The investigation into advanced MOF materials capable of generating both superoxide ions and monoclinic oxygen, compared to hydrogen peroxide as an oxidant, presents a fresh and effective strategy for mustard detoxification.

## 4. MOF Composites Degradation of Chemical Nerve Agents 

MOFs are typically found in powdered solid form and are challenging to process, which limits their potential application as protective layers. Electrospinning equipment is simple, the process is controllable, the cost is low, the spinning matrix is diverse, and it has broad application prospects in the fields of biomedical application, catalysis, and protective filtration. The preparation of catalyst-supported functional nanofibers by electrospinning combined with MOFs is an effective method to prepare materials with degradation protection. By electrospinning and in situ hydrolysis of metal salt, MOFs can be induced to grow uniformly on a cellulose surface via the reaction of solution heat, and a nanofiber membrane with a tight core–shell structure can be obtained. These compounds can effectively degrade nerve agents. MOFs and protective layers can be integrated through various methods such as coating, impregnation, and functionalization. By modifying MOFs into the protective layer, these materials can directly interact with and adsorb nerve agents, triggering catalytic reactions that effectively degrade toxic substances. Therefore, achieving uniform modification of functional MOF particles into flexible fiber substrates is a crucial step toward applying MOFs in protective masks and clothing. Functionalized MOF materials offer several advantages for efficient degradation. Unlike traditional organic degradants, they exhibit improved stability, recyclability, high porosity, and surface area, enhancing their degradation efficiency. Different combinations of metal clusters and organic ligands provide a wide range of tunability for adapting MOF materials to specific applications. Thus, the optimal design and synthesis of MOF materials can be tailored for specific pollutants to increase selectivity and efficiency.

### 4.1. MOF Composite Degradation of Chemical Nerve Agent Simulants

Selecting appropriate MOFs ensures effective catalytic activity under required operating conditions while maintaining stability and repeatability. For instance, cotton and polymer substrates have gained significant attention in recent years. Electrospinning technology has been proven suitable for preparing high-performance composite materials composed of functional MOFs and a range of polymers. One of the key challenges in this field is combining MOFs with polymers to achieve a uniform thickness and a continuous surface. Nanocellulose is renewable and has great potential for use as a support substrate, especially in cellulose aerogels and sponges. In 2019, Sui et al. developed a UiO-66-NH_2_-loaded cellulose sponge, a functional sponge loaded with MOFs in situ, which can achieve rapid degradation of the nerve agent mimicking agent DMNP with a half-life as short as 9 min [74]. This sponge has high porosity (88%), a high specific surface area (310.5 g/m^2^), and a low density (36 mg/cm^3^). Chemical cross-linking of UiO-66-NH_2_ powders on cellulose sponges helps to overcome the challenges encountered in the practical application of MOF powders. The synthesis of TiO_2_ atomic layer deposition (ALD) and solvothermal deposition MOFs has become one of the most adaptable growth technologies. 

**Figure 11:**
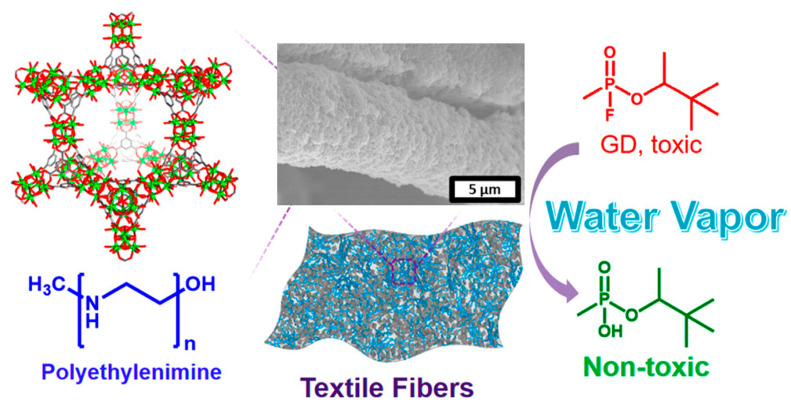
Schematic illustration of the synthetic routine of MOF-808/PEI/fibers used for GD degradation [75].

Although MOF powders can degrade organic phosphorus compounds, the practical application of this process is often hindered by the need for large amounts of water and volatile organic base solutions. In 2019, Farha et al. reported a promising and convenient method in which MOFs and non-volatile polymer bases were integrated into textile fibers to hydrolyze neurotoxic agents. This integration significantly reduced the size of the filters and improved the efficiency of protective clothing, as shown in Figure 11. This work provides a reference solution for degrading these harmful chemicals in real-world environments, offering a new approach to the design and preparation of protective materials [75].

**Figure 12:**
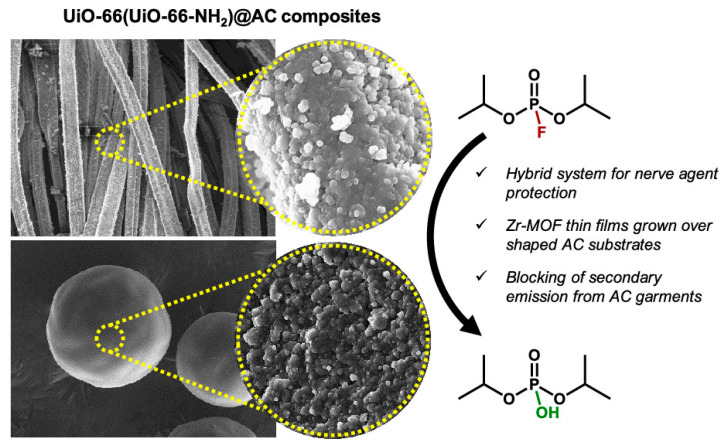
The porous structure of Zr-MOF/polymers [76].

In 2021, Navarro et al. achieved a controllable synthesis of representative zirconium-based MOFs thin films, [Zr_6_O_4_(OH)_4_(benzene-1,4-dicarboxylate-2-X)_6_], using a layer-by-layer method on the surface of carbonized substrates [76]. The resulting composite material exhibited a uniform distribution of MOF crystals on the outer surface of the porous structure and demonstrated rapid capture capability for diisopropyl fluorophosphate (DIFP), as shown in Figure 12. Compared to in situ MOF synthesis methods, the layer-by-layer assembly technique offers advantages by enabling homogeneous dispersion of Zr-MOFs on activated carbon surfaces, thus showcasing potential applications in chemical protective equipment.

Zr-MOFs with Lewis acidic sites are highly efficient catalysts for the degradation of organophosphorus nerve agents. However, their practical application is challenged by the use of volatile alkaline solutions in conjunction with Zr-MOF catalysts. The design of base heterogenization by Farha et al. is crucial for the practical application of MOFs as catalytic filters in decomposing chemical warfare agents [77]. Using the zirconium-based MOF NU-901, hydrolysis experiments were conducted on nerve agent simulants in the presence of different amine bases, with a half-life of less than 2 min. By utilizing low-volatility branched polymers and dendritic polymers, NU-901 exhibited catalytic performance comparable to that achieved using ethyl morpholine as a catalyst. In 2020, Barry. et al. reported a composite polymer material capable of adsorbing nerve agents within porous polymers until complete hydrolysis by MOF catalysts occurs, minimizing the use of multiple reagents to achieve maximum VX degradation rates exceeding 95% within 11 days [78].

**Figure 13:**
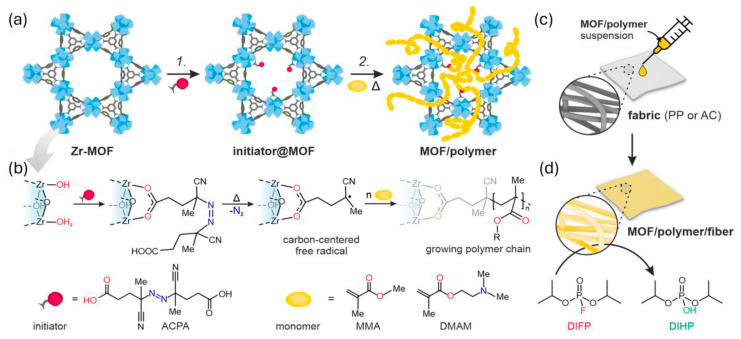
Schematic illustration of the mixture of MOF/polymer hybrids in the decomposition of diisopropyl fluorophosphate (DIFP). (**a**) The synthetic procedure of MOF/polymer incorporating ligands with the assistance of solvents, (**b**) the synthesis process of SALI (Single Atom Layered Isomer) utilizing 4,4′-azobis(cyanovaleric acid) and Zr-nodes to generate carbon-centered radicals, (**c**) Coating MOF/polymer hybrids onto fibers using the drop-casting method, and (**d**) the hydrolysis of DIFP (diisopropyl fluorophosphate) with MOF/polymer/fiber composite [80].

In 2020, Glover et al. utilized the ionic liquid-modified MOF UiO-66-NH_2_ to coat cotton, and the fabric’s PXRD characterization data closely matched that of UiO-66-NH_2._ This nanofiber exhibited the ability to degrade DMNP [79]. In 2023, Bury’s research team successfully synthesized a hybrid material consisting of MOFs and polymers by precisely controlling their interaction, resulting in improved processing performance. They also demonstrated that this complex displays high activity against the nerve agent DIFP [80], as depicted in Figure 13.

In 2021, Farha’s group developed a Zr-MOF/hydrogel composite catalyst that rapidly degrades organophosphorus compounds at room temperature. By coating MOF-808 hydrogel onto textiles, the combination of crosslinked polyethyleneimine (PEI)-based hydrogel and MOFs provided catalytically active Lewis acidic sites and the alkaline conditions required for catalyst regeneration, resulting in the fastest degradation rate achieved to date for MOF-based composite materials. This facile processing method for powder and fiber composites offers possibilities for the large-scale production of MOF-based protective equipment such as masks and clothing [81]. 2D metal-organic framework (MOF) nanoplates offer the advantage of possessing a higher number of degradation catalytic sites and lower diffusion barriers. However, achieving uniform vertical growth of ultra-thin 2D MOF nanoplates on fabrics presents a significant challenge. In 2024, Wang’s team utilized a microwave-assisted solvothermal method to grow ultra-thin 2D Zr-BTB metal-organic framework (MOF) nanoplates on PP fibers. This approach addresses challenges in achieving uniform vertical growth of these nanoplates on fabrics. The fabricated material exhibited exceptional catalytic efficacy in the nerve agent simulant DMNP both in solution and on contaminated surfaces. The potential applications of PP/MOF nanoplate composite fabric in chemical protection and environmental remediation render it a promising choice for environmental purification and protective clothing against chemical hazards [82].

### 4.2. MOF Composite Degradation of Mustard Gas Simulants

**Figure 14:**
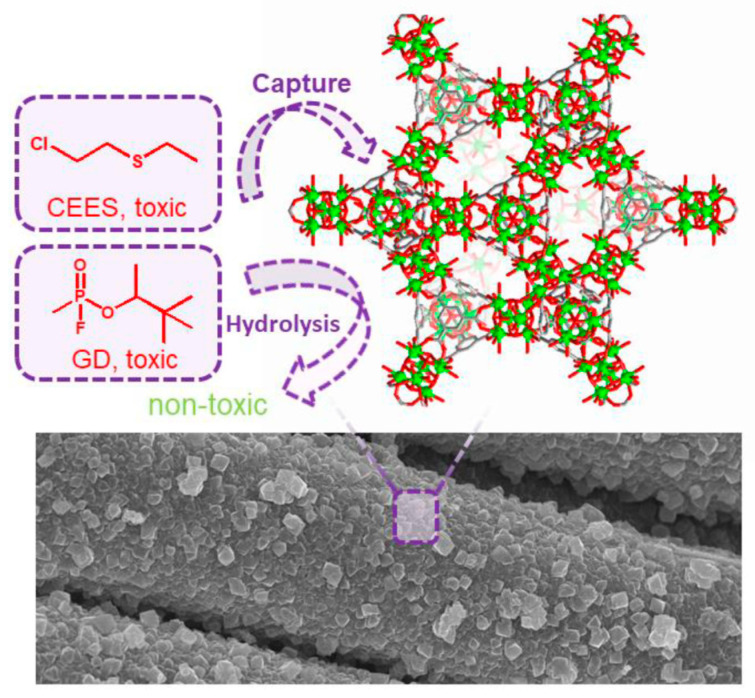
Illustration of the MOF/fibers used for 2-chloroethyl ethyl sulfide (CEES) capture and soman (GD) detoxification [83].

Enhancing the performance of MOFs in the MOF–textile format necessitates the development of novel MOF materials and synthetic strategies to effectively catalyze the purification of toxic chemicals, addressing a crucial need in this field. In 2019, Farha et al. reported an innovative and template-free synthesis strategy for fabricating textile-based zirconium MOFs. Using a simple process of immersion and deposition, the researchers introduced zirconium ions and organic ligands onto the surface of the textile, followed by a one-step hydrothermal reaction to form a coating of MOF, as shown in Figure 14. The advantage of this method lies in its scalability, allowing for coating of large-area textiles. Among all reported MOF/fiber composite materials, MOF-808/fibers exhibited higher rates of nerve agent hydrolysis. Furthermore, these highly porous composites demonstrated prolonged protection against the mustard gas simulant CEES [83]. An additional benefit of this synthetic approach is that it eliminates the need for cumbersome pre-treatment steps on fibers and enables direct synthesis of MOF coatings on various types of fiber surfaces.

Zr-MOFs exhibit promising potential for the degradation of neurochemical warfare agents, but their catalytic performance against blister agents remains limited. Furthermore, challenges such as powder handling issues and inadequate adsorption capacity hinder the practical application of MOFs in detoxification processes. In 2022, Tao et al. successfully synthesized a series of defective granular UiO-66-NH_2_ metal–organogels by precisely adjusting the addition of concentrated hydrochloric acid to eliminate contamination from 2-CEES [84]. Remarkably, these defective granular UiO-66-NH_2_ metal–organogels exhibited an unprecedented half-life reduction for purified 2-CEES under ambient conditions as low as 7.6 min, representing the highest reported value among MOFs. The decontamination mechanism involved a substitution reaction between the amino group on the linker in UiO-66-NH_2_ and 2-CEES. This product reduced its toxicity and enhanced its stability and reusability, as confirmed by recovery tests. The UiO-66-NH_2_ dry gel exhibited a remarkable 2-CEES vapor adsorption capacity of 802 mg/g. Its desorption capacity was only 28 wt% after seven days of exposure. These findings highlight the dual functionality of ultra-fast degradation and a high adsorption capacity, providing a solid foundation for its potential application as an advanced protective medium in the future.

Despite the existence of numerous reported catalysts exhibiting CWA detoxification capabilities, there remains a scarcity of effective strategies for seamlessly integrating these catalytically active materials into practical fabrics, thereby ensuring efficient protection against the simultaneous presence of multiple CWAs. In 2023, Jiao et al. employed a combination of electrostatic spinning and in situ solvent–thermal reactions to fabricate PAN@Zr(OH)_4_@MOF-808 nanofibrous membranes for the detoxification of DMNP and CEES [85]. The MOF-808 component catalytically facilitated the hydrolysis of DMNP with a short half-life of 1.19 min. MOF-808 achieved an adsorption efficiency of 89.3% for CEES removal within 12 h. The PAN@Zr(OH)_4_@MOF-808 nanofiber membrane exhibited superior CEES blocking efficacy compared to PAN@Zr(OH)_4_ nanofiber membranes. The preparation method also facilitated exceptional interfacial adhesion between the components, thereby contributing to the remarkable recycling stability of the PAN@Zr(OH)_4_@MOF-808 membrane. Consequently, these composite nanofabrics hold significant potential for practical applications and present a novel approach to constructing versatile protective detoxification materials. Zirconium-based metal materials exhibit promising potential for detoxifying CWAs owing to their exceptional stability and porosity. However, their practical application is impeded by powder form and solid-phase degradation. In 2023, Zhang et al. successfully synthesized granular MOF-808 metal–organogels (G808) for the catalytic degradation of 2-CEES under varying moisture conditions [86]. Moreover, the detoxification efficiency of CEES was notably augmented with increasing moisture content. The mechanism of catalytic degradation was elucidated through gas chromatography (GC), gas chromatography–mass spectrometry (GC-MS), and theoretical calculations. Furthermore, granular G808 exhibited reusability and adaptability in high-humidity environments, which has the potential to be an exceptional protective material with practical applications.

## 5. Conclusions

In recent years, functional metal–organic frameworks have gained significant attention for their application in the catalytic degradation of chemical warfare agents with the advantages offered by porous materials and polymer nanofibers. These MOFs possess Lewis acid sites, where the metal nodes also exhibit catalytic activity regarding the ester bond hydrolysis of nerve agents. One of the most attractive features of MOFs compared to traditional porous materials is the flexibility of their structure, which provides more material options in the degradation and adsorption of chemical warfare agents. Furthermore, modifications to the ligands of MOFs can influence their catalytic activity. Despite some MOFs demonstrating effective catalytic degradation capabilities, certain challenges still need to be addressed for practical implementation. For instance, buffer solutions and alkaline reagents like N-ethylmorphine are employed in the degradation of chemical warfare agents. Therefore, despite excellent catalytic properties exhibited by MOF materials, specific conditions are required for reactions that pose significant obstacles in practical applications such as gas masks or protective clothing. This review aims to provide novel insights for the development of new materials for MOF detoxification and the application of MOF composite materials in protective equipment. In recent years, most reported studies have primarily focused on detoxifying CWAs/mimics in the water phase rather than the gas phase. By combining MOFs with porous materials or polymer nanofibers, various synthesis strategies, materials, analytical tools, deposition methods, and interaction mechanisms are investigated to design a range of efficient filtration devices that can achieve CWA degradation in the gas phase. This review also presents advancements made in MOF composite research where composite materials demonstrate exceptional mechanical properties surpassing those of traditional single materials including higher strength, stiffness, and durability. As a current popular research direction, composite materials eliminate reliance on traditional buffer environments and serve as ideal candidates for personal protective equipment by offering excellent performance. By designing synthetic ligands, controlling pore size, and effectively integration with fiber materials, MOFs composites have shown great potential for the development of protective material against chemical warfare agents (CWAs).
